# Platelets are highly efficient and efficacious carriers for tumor-targeted nano-drug delivery

**DOI:** 10.1080/10717544.2022.2053762

**Published:** 2022-03-23

**Authors:** Qi-Rui Li, Hua-Zhen Xu, Rong-Cheng Xiao, Yan Liu, Jun-Ming Tang, Jian Li, Ting-Ting Yu, Bin Liu, Liu-Gen Li, Mei-Fang Wang, Ning Han, Yong-Hong Xu, Chao Wang, Naoki Komatsu, Li Zhao, Xing-Chun Peng, Tong-Fei Li, Xiao Chen

**Affiliations:** a School of Basic Medical Sciences, Hubei University of Medicine, Shiyan, China; b Hubei Key Laboratory of Embryonic Stem Cell Research, Taihe Hospital of Shiyan, Hubei University of Medicine, Shiyan, China; c Department of Pharmacology, School of Basic Medical Sciences, Wuhan University, Wuhan, China; d Hubei Provincial Key Laboratory of Developmentally Originated Disease, Wuhan, China; e Institute of Ophthalmological Research, Department of Ophthalmology, Renmin Hospital of Wuhan University, Wuhan, China; f Graduate School of Human and Environmental Studies, Kyoto University, Kyoto, Japan; g State Key Laboratory of Radiation Medicine and Protection, School of Radiation Medicine and Protection & School for Radiological and Interdisciplinary Sciences (RAD-X), Collaborative Innovation Center of Radiation Medicine of Jiangsu Higher Education Institutions, Soochow University, Suzhou, China

**Keywords:** Platelets, nano-drug, tumor-targeted delivery, doxorubicin, nanodiamonds

## Abstract

The present work aims to prove the concept of tumor-targeted drug delivery mediated by platelets. Doxorubicin (DOX) attached to nanodiamonds (ND-DOX) was investigated as the model payload drug of platelets. In vitro experiments first showed that ND-DOX could be loaded in mouse platelets in a dose-dependent manner with a markedly higher efficiency and capacity than free DOX. ND-DOX-loaded platelets (Plt@ND-DOX) maintained viability and ND-DOX could be stably held in the platelets for at least 4 hr. Next, mouse Lewis lung cancer cells were found to activate Plt@ND-DOX and thereby stimulate cargo unloading of Plt@ND-DOX. The unloaded ND-DOX was taken up by co-cultured cancer cells which consequently exhibited loss of viability, proliferation suppression and apoptosis. In vivo, Plt@ND-DOX displayed significantly prolonged blood circulation time over ND-DOX and DOX in mice, and Lewis tumor grafts demonstrated infiltration, activation and cargo unloading of Plt@ND-DOX in the tumor tissue. Consequently, Plt@ND-DOX effectively reversed the growth of Lewis tumor grafts which exhibited significant inhibition of cell proliferation and apoptosis. Importantly, Plt@ND-DOX displayed a markedly higher therapeutic potency than free DOX but without the severe systemic toxicity associated with DOX. Our findings are concrete proof of platelets as efficient and efficacious carriers for tumor-targeted nano-drug delivery with the following features: 1) large loading capacity and high loading efficiency, 2) good tolerance of cargo drug, 3) stable cargo retention and no cargo unloading in the absence of stimulation, 4) prolonged blood circulation time, and 5) excellent tumor distribution and tumor-activated drug unloading leading to high therapeutic potency and few adverse effects. Platelets hold great potential as efficient and efficacious carriers for tumor-targeted nano-drug delivery.

## 1. Introduction

Platelets are discoid anucleate fragments of megakaryocytes and considered the smallest hemopoietic cells in the blood circulation (Davizon-Castillo et al., 2020; Machlus & Italiano, 2013; Lefrançais & Looney, 2019). The critical roles of platelets have long been recognized in hemostasis, thrombosis, tissue regeneration and wound repair (Holinstat, 2017; Koupenova et al., 2018). Recently, platelets have been revealed to have complex interactions with cancer cells and stromal cells and play vital roles in the genesis and progression of malignant tumors. Malignant tumors are known to induce platelet production and cancer patients frequently exhibit elevated platelet counts which are associated with increased incidence of venous thromboembolism (Suzuki-Inoue, 2019; Key et al., 2020). The ever increasing metabolic demand of tumor growth requires constant angiogenesis i.e. the formation of new blood vessels. Platelets supply angiogenic factors to promote tumor angiogenesis and are needed to maintain the functional integrity of newly formed blood vessels (Yan et al., 2014; Haemmerle et al., 2018; Li et al., 2019; Lu et al., 2019; Combes et al., 2020; Li et al., 2020; Miao et al., 2020). Platelets can be recruited to the tumor microenvironment (TME) to bolster cancer proliferation through release of mitogenic factors. Platelets also act in concert with other immune cells in the TME to create an immunosuppressive microenvironment that promotes therapy resistance (D'Alessandro et al., 2015; Huijbers et al., 2016; Schlesinger, 2018; Xu et al., 2018; Jia et al., 2020). Moreover, platelets can form aggregates with metastatic cancer cells in the blood stream and thereby protect the cancer cells from the attack by blood shear forces and circulating immune cells. In light of the significant roles of platelets in tumor pathophysiology, there has been a mounting interest in targeting and exploiting platelets for development of effective anti-tumor therapeutic strategies.

Tumor-targeted drug delivery is highly desirable for tumor therapy and platelets have emerged as intriguing candidate drug carriers to meet this need (Li et al., 2021). Rationales for the strategy of platelet-mediated tumor drug delivery includes 1) platelets are recruited by tumors to maintain blood vessel integrity; 2) platelets are capable of endocytosis and secretion (Banerjee et al., 2020; Golebiewska & Poole, 2015), which is amenable to drug loading and unloading; 3) platelets are able to infiltrate in the TME; and 4) platelets can be easily isolated from the blood in large quantities and platelet transfusions are commonly performed in the clinic. Taking advantage of these properties, a few tumor drug delivery devices based on platelets have emerged (Sarkar et al., 2013). Lv et al., 2016; Xu et al., 2017; Notwithstanding, the picture of platelet-mediated drug delivery is far from complete and this strategy needs more substantiation and characterization. To this purpose, the present study was carried out using doxorubicin (DOX), an anti-tumor chemotherapeutic agent, reversibly attached to nanodiamonds (ND-DOX), as the drug model to be delivered by platelets. Free DOX was also used as a cargo drug for comparison. We have previously used ND-DOX to demonstrate tumor drug delivery mediated by immune cells such as monocytes, macrophages and dendritic cells (Li et al., 2017, 2018; Wang et al., 2018). Thus, a second purpose of the current work is to characterize platelets, being anucleate fragmented cells, for drug delivery carriers in light of the findings on those nucleate whole cells. In vitro experiments were first performed to assay drug loading and unloading, effects of drug loading on platelet activation and viability, cargo drug transfer from platelets to co-cultured cancer cells and cargo drug toxicity to the cancer cells. In vivo experiments were next carried out on mice to evaluate blood drug clearance and to confirm tumor-targeted platelet-mediated drug delivery whose therapeutic efficacy, systemic toxicity and vital organ toxicity were finally evaluated. Discussion is made highlighting some original and significant findings, with particular respect to previously reported drug delivery tactics based on platelets and other cells.

## 2. Materials and methods

### 2.1. Nanodiamond-doxorubicin conjugates (ND-DOX)

**Figure 1.**
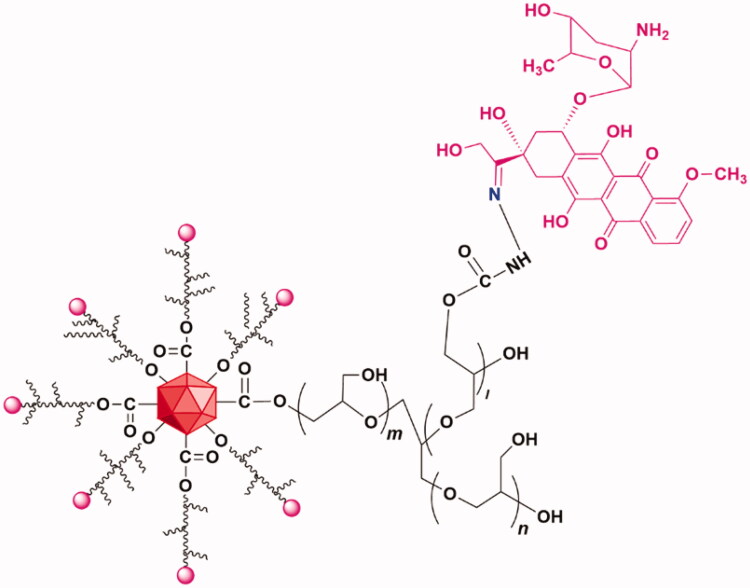
Structural composition of ND-DOX. DOX was conjugated with polyglycerol-coated nanodiamond via the hydrazone linkage.

ND-DOX particles were fabricated from nanodiamonds (ND) (4–5 nm in diameter) with a surface coating of polyglycerol (PG). DOX was loaded via the acid-sensitive hydrazone linkage to the PG coating giving the ND-DOX particles which have an aqueous hydrodynamic diameter of 83.9 ± 32.3 nm with good solubility in physiological solutions. The synthesis, physical and chemical characterization, and particularly the pH-sensitive release of DOX from ND-DOX have been detailed in our previous work (Zhao et al., 2014; Wang et al., 2018). Further our previous work has also demonstrated that the lysosomal compartment is the major deposit site for internalized Nano-DOX and release of DOX from internalized Nano-DOX a slow process, which is usually not significant until 40 h post cell uptake (Gresele et al., 2017). The chemical structure of ND-DOX was shown in Figure 1. ND-DOX solution was kept in water at 4 °C and was sonicated in a water bath for 3 min before being diluted with culture medium into working concentrations. All concentrations and dosages of ND-DOX were normalized to DOX.

### 2.2. Cell models

Preparation and characterization of mouse platelets (Plt) were performed according to published protocols (Xu et al., 2017; Ying et al., 2018). Briefly, peripheral blood extracted from female C57BL mice (5–6 weeks of age, 22 ∼ 24 g) under nembutal anesthesia was placed in an anticoagulant tube and centrifuged at 200 g for 15 min at room temperature to obtain platelet-rich plasma. The platelet-rich plasma was further centrifuged at 100 g for 15 min for thorough removal of red blood cells (Figure S1). The resultant supernatant was centrifuged at 800 g for 15 min to obtain the platelets. Mouse Lewis lung cancer cells (LLC) were purchased from the Cell Bank of Shanghai Institutes for Biological Sciences (Shanghai, China). Cell cultures were performed using DMEM medium (Sigma-Aldrich, St Louis, USA) supplemented with 10% fetal bovine serum (QmSuero/Tsingmu Biotechnology, Wuhan) in a humidified incubator (5%CO_2_/95% air atmosphere at 37 °C).

### 2.3. Drug uptake and release by platelets

For assay of time-dependent drug uptake, platelets in EP tubes with a density of 1 × 10^6^/100 uL, 300 uL per tube were incubated with 1 μg/mL of ND-DOX or DOX in culture medium at 37 °C. One tube of drug-loaded platelets was taken at different time points for flow cytometry analysis of intra-platelet drug-derived fluorescence (cytoflex, Beckman Coulter, USA). For assay of drug loading capacity, platelets in EP tubes were incubated with 1 μg/mL of ND-DOX or DOX in culture medium at 37 °C for 4 hr and drug-loaded platelets were taken out by centrifugation for flow cytometry analysis. The resultant supernatants were analyzed by fluorescence spectrophotometry (SpectraMax i3, Molecular Devices) for drug loading efficiency. Drug-loaded platelets were also fixed with 4% of paraformaldehyde and then observed with a laser scanning confocal microscope (FV3000RS, Olympus, Hubei University of Medicine). Drug-loaded platelets (1 μg/mL of ND-DOX or DOX, 37 °C, 4 hr) were incubated with fresh medium in EP tubes at 37 °C. One tube of platelets was taken at different time points for flow cytometry analysis. Drug-loaded platelets in petri dishes were also observed with a high-content cytometer (Operetta CLS, Perkin Elmer).

### 2.4. Viability, cell membrane integrity and cell death of drug-loaded platelets

Drug-loaded platelets (1 μg/mL of ND-DOX or DOX, 37 °C, 4 hr) were transferred to 96-well plates and incubated with CCK-8 for 2 hr at 37 °C before absorbance at 450 nm was measured with a plate reader (SpectraMax i3, Molecular Devices). For determination of cell membrane integrity, drug-loaded platelets in culture medium were incubated with 10 μg/mL of fluorescein diacetate (FDA) at room temperature for 5 min before being observed with a laser scanning confocal microscope. For assay of apoptosis and autophagy, total proteins from drug-loaded platelets were extracted for western blotting analysis of caspase-3, ATG5, Beclin-1 and LC3-II. Samples of drug-loaded platelets were also prepared and observed with transmission electron microscopy (TEM, HT7700, Hitachi, Japan).

### 2.5. Co-culture of ND-DOX-loaded platelets with lung cancer cells

ND-DOX-loaded platelets were co-cultured with Lewis cells in 24-well plates for 2 hr. The co-culture was then observed with a microscope. The platelets were next taken out for measurement of aggregation by dynamic light scattering (DLS, Nano ZS90, Malvern Instruments, UK) and surface P-selectin expression was analyzed by immunofluorescent staining and flow cytometry. Alternatively, ND-DOX-loaded platelets on cover glasses were incubated with Lewis cell-conditioned culture medium for 2 hr before being observed with scanning electron microscopy (SEM). For assay of drug transfer from ND-DOX-loaded platelets to Lewis cells, ND-DOX-loaded platelets were incubated with Lewis cells at 37 °C for 2 hr in 24-well plates. The mixture was then taken out for flow cytometry analysis of ND-DOX-derived fluorescence in the platelets and Lewis cells. Alternatively, Lewis cells grown on cover glasses were incubated with ND-DOX-loaded platelets for 2 hr before Hoechst 33342 staining and confocal microscopy. For assay of Lewis cell viability in co-culture, Lewis cells were incubated with ND-DOX-loaded platelets in 96-well plates for 12 hr before being subjected to CCK-8 test. For assay of cell proliferation, Lewis cells were incubated with ND-DOX-loaded platelets in 6-well plates for 12 hr and total proteins were extracted for western blotting analysis of PCNA and CDK4. For assay of apoptosis, Lewis cells were incubated with ND-DOX-loaded platelets in 24-well plates for 12 hr before cell surface expression of Annexin-v (Purchased from CHAMOT BIOTECHNOLOGY CO., LTD.) was analyzed by immunofluorescent staining and flow cytometry. The numbers of Lewis cells *vs* ND-DOX-loaded platelets was 1 × 10^4^: 5 × 10^4^ per well in 96-well format, 2 × 10^5^: 5 × 10^5^ per well in 24-well format, and 1 × 10^6^: 5 × 10^6^ per well in 6-well plates.

### 2.6. Immunofluorescent staining, flow cytometry and Western blotting

Cellular fluorescence was acquired on a Beckman Cytoflex flow cytometer. DOX or Nano-DOX fluorescence was acquired in the PE channel. At least 1 × 10^4^ cells/per sample were acquired for every collection. Geometric means (GM) were used to quantify the mean fluorescent intensity (MFI). For western analysis, platelets treated as required were rinsed twice with PBS and lysed in RIPA buffer with 1% protease inhibitor. Cell lysates were centrifuged and protein concentration was measured using a BCA assay kit. Equal protein aliquots (10 μg) were fractionated by SDS-PAGE and transferred to a PVDF membrane. The membranes were blocked with 3% bovine serum albumin in TBST and incubated with antibodies against CD42b (11026-1-AP, Proteintech, Wuhan, China), Caspase-3 (50599-2-Ig, Proteintech, Wuhan, China), CDK4 (11026-1-AP, Proteintech, Wuhan, China), PCNA (bs-2007R, Bioss, Beijing, China), and GADPH (PMK053C, BioPM, Wuhan, China) at 4 °C overnight. The membranes were then incubated with horseradish peroxidase-conjugated secondary antibody before protein bands were developed using a ECL. The films were exposed using a Bio Imaging system (170-8265, Bio-Rad).

### 2.7. In vivo animal experiments

For evaluation of blood drug concentration, female C57BL mice at 4–5 weeks of age (14 ∼ 18 g) were given a bolus injection of Plt@ND-DOX, ND-DOX, or DOX via the caudal vein (0.1 mg/kg bw). Blood samples from the retro-orbital plexus were then drawn at different time points and placed in a 96-well-plate for fluorescence spectrophotometry analysis of drug concentration standardized to DOX. Excitation wavelength was 488 nm and emission wavelength 590 nm. For therapeutic efficacy and toxicity study, female C57BL mice at 4–5 weeks of age (14 ∼ 18 g) were each subcutaneously injected at the right armpit with Lewis lung cancer cells (1 × 10^6^ cells/200 μL of PBS). The animals were randomly grouped (5 mice per group) when the tumor growth reached about 500 mm^3^. Injections of PBS, Plt, Plt@ND-DOX, ND-DOX, DOX, each in 200 μL of PBS per mouse, were then given 3 times through the caudal vein at an interval of 24 hr. Plt@ND-DOX were prepared by loading 300 × 10^6^ platelets with 1 μg/mL of ND-DOX for 4 hr. Dosages were 0.1 mg/kg bw for Plt@ND-DOX and ND-DOX, 0.1 and 5 mg/kg bw for DOX. Body weight and tumor volume were monitored on a daily basis throughout the experiment period. At 24 hr after the last injection, the animals were sacrificed and tumor grafts, vital organs were harvested. Expression of CD42b, CD41, CD62p, CDK4, PCNA and BAX in the tumor tissues were examined by immunohistochemical staining (IHC). Drug distribution in tumor tissues and vital organs was examined through ex vivo imaging and fluorescent microscopy of tissues sections. HE staining was also performed on tissue sections of vital organs. The animals were purchased from the Experimental Animal Center of Hubei University of Medicine (Shiyan, China) and housed in a temperature-controlled environment with fresh water and rodent diet available at all times. All animal handling and experimental procedures were in line with protocols approved by the Animal Care Committee at the Hubei University of Medicine.

### 2.8. Tissue staining

Briefly, tumor tissues and vital organs were fixed and embedded, then ultra-thin sections (5 μm) are performed. For HE staining, paraffin sections were dewaxed, rehydrated. Hematoxylin was used to stain the nucleus and eosin was used to label the cytoplasm. For immunohistochemistry, paraffin sections were dewaxed, rehydrated, and antigens were repaired by incubation with sodium citrate for 20 min, before further incubation with 3% hydrogen peroxide for 10 min at room temperature. The paraffin sections were blocked with 5% BSA for 30 min, stained with primary antibodies of Bax (50599-2-Ig, Proteintech, Wuhan, China), CDK4 (11026-1-AP, Proteintech, Wuhan, China), PCNA (bs-2007R, Bioss, Beijing, China), CD42b (bs-2347R, Bioss, Beijing, China), CD 41 (bs-2636R, Bioss, Beijing, China) and CD62p (bs-0561R, Bioss, Beijing, China) at 4 °C overnight, then washed with PBS, and finally stained with secondary antibody (PV-9000, ZSGB-BIO, Beijing, China) for 1 hr at 37 °C. Diaminobenzidine (DAB, ZLI-9018, ZSGB-BIO, Beijing, China) was applied for coloration for 5 min at room temperature. Finally, the sections were imaged using an Orthomorphic microscope (BX53, Olympus, Japan). For fluorescent staining, paraffin sections were dewaxed, rehydrated and DAPI (C1002, Beyotime, Shanghai, China) was applied to label the nucleus for 3 min.

### 2.9. Statistical analysis

Statistical differences between groups were analyzed using One-way analysis of variance (ANOVA).

## 3. Results

### 3.1. Nd-DOX were stably loaded in mouse platelets

**Figure 2.**
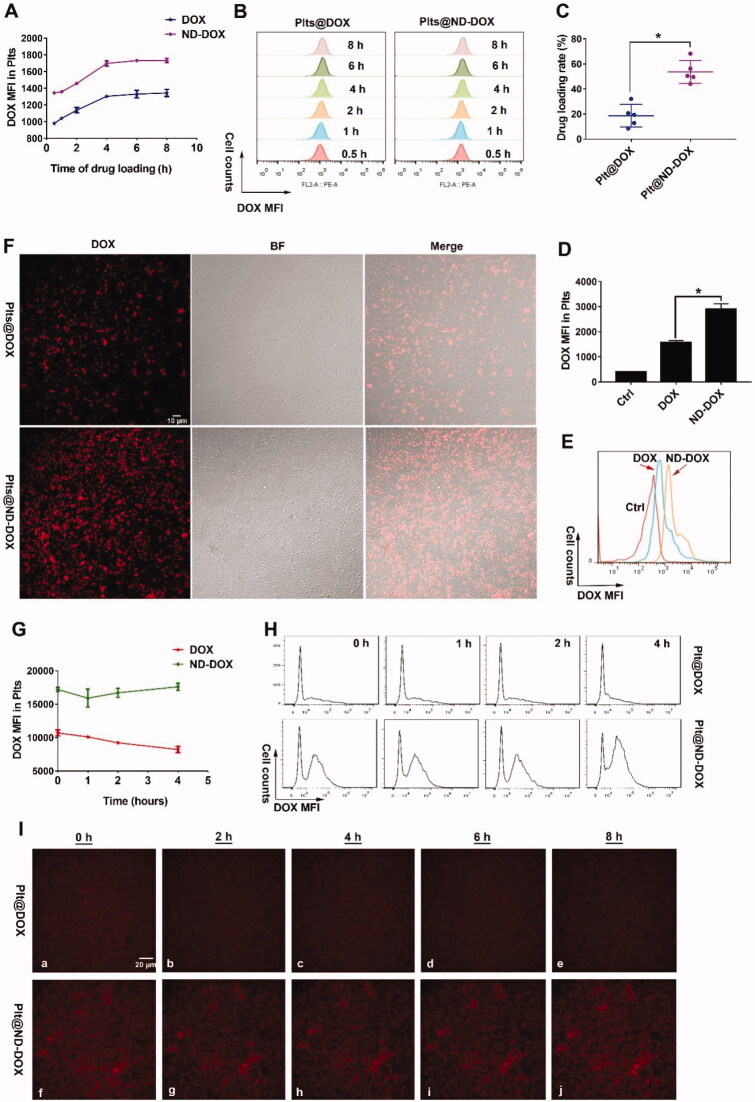
Loading and retention of ND-DOX by platelets. (A,B) Time-dependent uptake of ND-DOX and DOX by platelets assayed by flow cytometry. (C) Loading efficiency of ND-DOX and DOX by platelets determined by fluorescence spectrophotometry. (D,E) Loading capacity of ND-DOX and DOX by platelets assayed by flow cytometry. (F) Fluorescent microscopy of platelets loaded with ND-DOX or DOX. (G,H) Cargo unloading of platelets loaded with ND-DOX or DOX assayed by flow cytometry. (I) Time-lapse observation of platelets loaded with ND-DOX or DOX. Geometric means were used to quantify the mean fluorescence intensity (MFI) of flow cytometry. Values were means ± SD (*n* = 3, *p < .05). Plt@ND-DOX refers to platelets loaded with ND-DOX; Plt@DOX refers to platelets loaded with DOX.

Both ND-DOX and DOX displayed a time-dependent uptake by in vitro mouse platelets and saturation was reached after 4 hr of incubation with culture medium containing 1 μg/mL of ND-DOX or DOX (Figure 2(A,B)). Mouse platelets had a markedly higher loading efficiency (Figure 2(C)) and capacity (Figure 2(D,E)) for ND-DOX than DOX when the platelets had been incubated with 1 μg/mL of ND-DOX or DOX for 4 hr. Fluorescent microscopy also confirmed higher ND-DOX uptake than DOX under the same condition (Figure 2(F)). Quantitative analysis revealed that ND-DOX-loaded platelets could stably held their cargo for at least 4 hr in fresh culture medium while DOX-loaded platelets exhibited obvious cargo unloading over 4 hr (Figure 2(G,H)), which was corroborated by time-lapse microscopy (Figure 2(I)).

### 3.2. Nd-DOX-loaded platelets maintained viability

**Figure 3.**
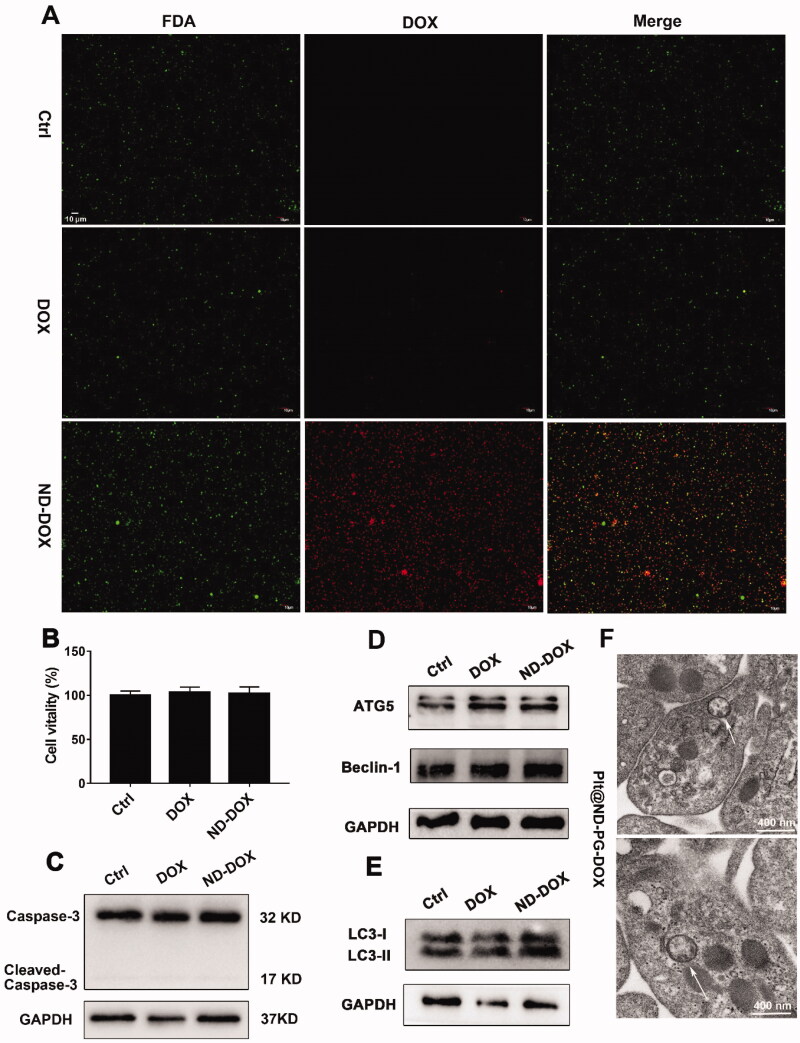
ND-DOX-loaded platelets maintained viability probably through induction of autophagy. (A) Microscopy of FDA staining of platelets loaded with ND-DOX or DOX. (B) Viability of platelets loaded with ND-DOX or DOX, assayed by CCK-8 test. (C–E) Western blot analysis of caspase-3, ATG5, Beclin-1 and LC-3 expression in platelets loaded with ND-DOX or DOX. (F) TEM observation of platelets loaded with ND-DOX (Plt@ND-DOX). White arrow marked was autophagosomes. Values were means ± SD (*n* = 3, * *p* < .05). Plt@ND-DOX refers to platelets loaded with ND-DOX.

Both ND-DOX- and DOX-loaded mouse platelets maintained their cell membrane integrity and viability as indicated by FDA staining, CCK-8 assay, and caspase-3 expression assay (Figure 3(A–C)). But ND-DOX induced significant autophagy in the platelets as indicated by the upregulated markers of autophagy activation such as ATG5, Beclin-1, LC3-II and most importantly, the appearance of autophagosomes in the cytoplasm (Figure 3(D–F)). DOX-loaded platelets also showed signs of autophagy activation (Figure 3(D,E)).

### 3.3. Tumor cells activated ND-DOX-loaded platelets to release ND-DOX which then repressed the tumor cells

**Figure 4.**
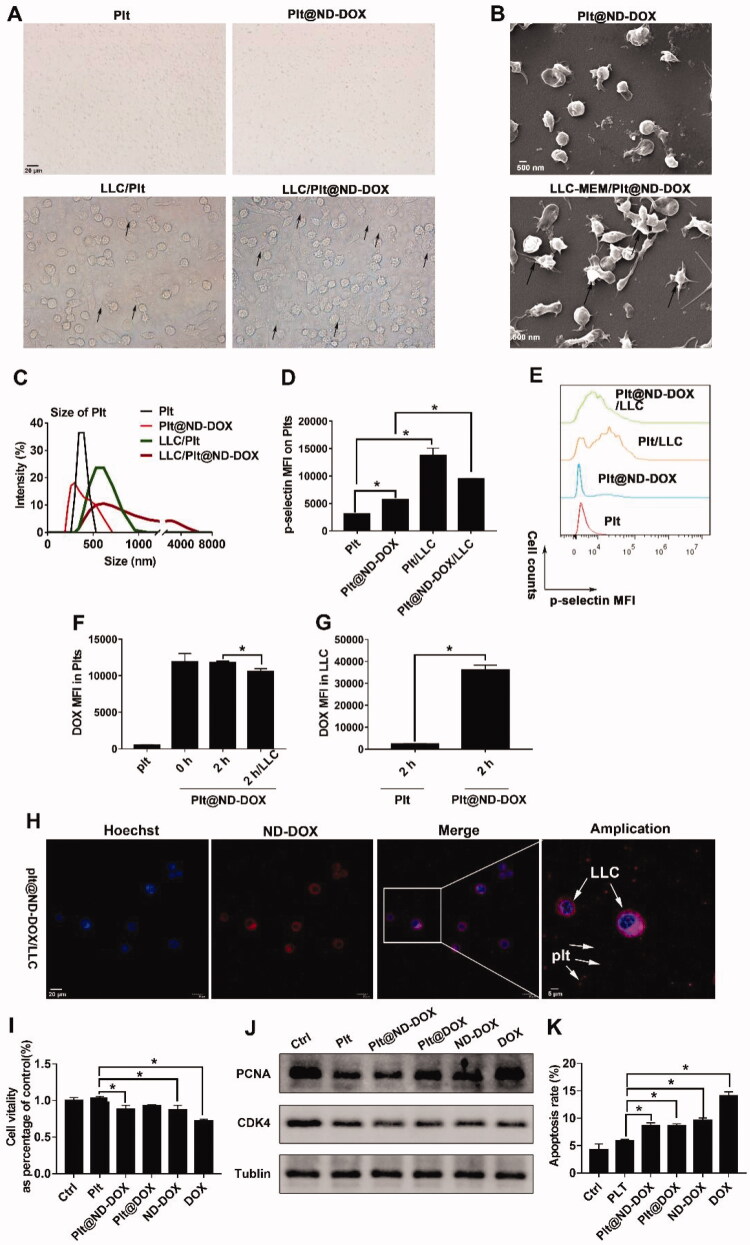
Lewis lung cancer cells (LLC) activated ND-DOX-loaded platelets to release ND-DOX which entered and damaged the cancer cells. (A) Microscopy of co-cultured Lewis cells and platelets loaded with ND-DOX or DOX. Arrow marked were platelet aggregates. (B) TEM observation of ND-DOX-loaded platelets in fresh culture medium or cancer cell-conditioned culture medium. Arrow marked were pseudopodia from activated platelets. (C) Aggregation of platelets and ND-DOX-loaded platelets in the presence and absence of the cancer cells. Assay was dynamic light scattering assay. (D,E) Surface expression of P-selectin in platelets and ND-DOX-loaded platelets in the presence and absence of the cancer cells. (F) Unloading of ND-DOX by ND-DOX-loaded platelets after 2-hr co-culture with cancer cells. Detection was by flow cytometry. (G) Cancer cell uptake of ND-DOX released from ND-DOX-loaded platelets in co-culture for 2 hr. Detection was by flow cytometry. (H) Fluorescent microscopy of ND-DOX-loaded platelets and cancer cells after 2-hr co-culture. (I) Viability of cancer cells after 12-hr co-culture with empty platelets, ND-DOX-loaded platelets or DOX-loaded-platelets. ND-DOX and DOX treatments were given as controls. (J) Expression of PCNA and CDK4 in cancer cells after 12-hr co-culture with empty platelets, ND-DOX-loaded platelets or DOX-loaded-platelets. ND-DOX and DOX treatments were given as controls. (K) Apoptosis of cancer cells after 12-hr co-culture with empty platelets, ND-DOX-loaded platelets or DOX-loaded-platelets. ND-DOX and DOX treatments were given as controls. Apoptosis was detected by annexin-v staining and flow cytometry. Geometric means were used to quantify the mean fluorescence intensity (MFI) of flow cytometry. Values were means ± SD (*n* = 3, *p < .05). Representative flow cytometry raw data were shown in Figure S2 of the supporting information. Plt@ND-DOX refers to platelets loaded with ND-DOX; Plt@DOX refers to platelets loaded with DOX.

Cancer cells are reported to activate platelets (Catani et al., 2020; Plantureux et al., 2020) and activated platelets are known to release granules including dense granules, α-granules, and lysosomes (Harrison & Cramer, 1993; Zuo et al., 2019). Thus, we had supposed that lung cancer cells can activate ND-DOX-loaded platelets to release ND-DOX likely sequestered in the autophagosomes. As expected, under the influence of the Lewis lung cancer cells (in co-culture or in conditioned culture medium), mouse platelets, either ND-DOX-loaded or not, exhibited characteristic signs of activation including aggregation (Figure 4(A–C)), formation of pseudopodia (Figure 4(B)), and increased surface presence of P-selectin (Figure 4(D,E)). Notably, ND-DOX per se only caused insignificant activation of platelets (Figure 4(A–E)). Subsequently, ND-DOX-loaded platelets released payload drug into co-cultured Lewis cells (Figure 4(F,G)) and fluorescent microscopy confirmed the drug internalized by the cancer cells to be ND-DOX as indicated by the cytoplasmic fluorescence (Figure 4(H)). We have previously demonstrated that ND-DOX mainly stays in the cytoplasm while free DOX mostly goes into the nucleus (Li et al., 2017, 2018). The cancer cells with internalized ND-DOX then exhibited decreased viability, growth suppression and apoptosis (Figure 4(I–K)).

### 3.4. Nd-DOX-loaded platelets displayed markedly slowed blood clearance and infiltrated in tumor tissue and were activated to release ND-DOX

**Figure 5.**
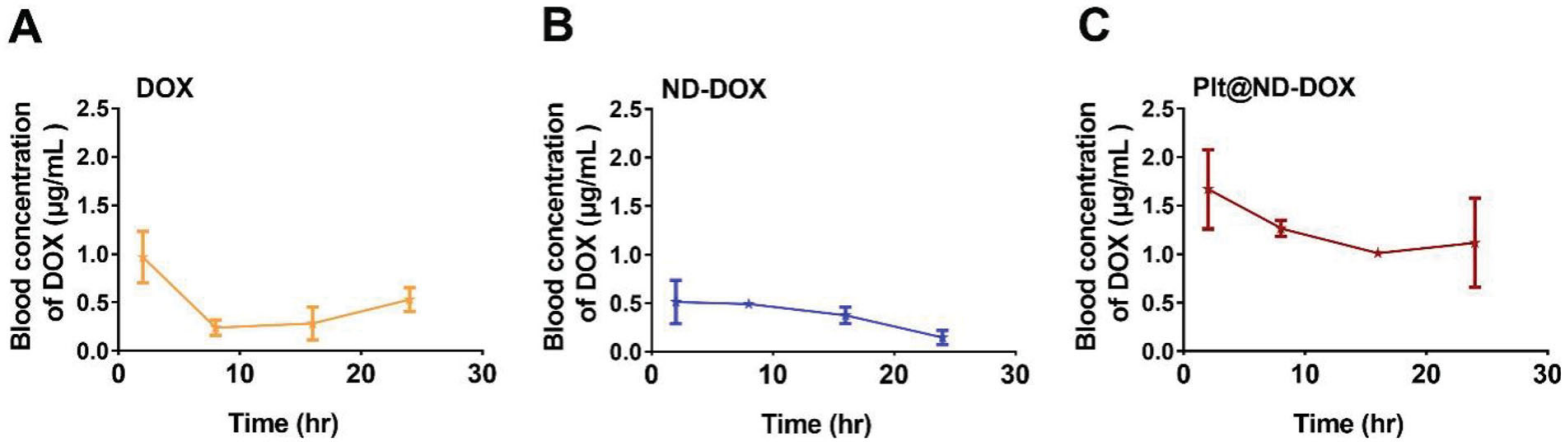
Blood concentrations of Plt@ND-DOX, ND-DOX and DOX within 24 hr of an intravenous bolus injection (normalized to 0.1 mg/kg bw of DOX). Blood samples from the retro-orbital plexus were drawn at hr 2, 8, 16, and 24 for fluorescence spectrophotometry analysis of drug concentration standardized to DOX. Values were means ± SD (*n* = 3, * *p* < .05).

In vivo experiments were conducted first to evaluate blood clearance of Plt@ND-DOX against ND-DOX and DOX after an intravenous bolus injection (normalized to 0.1 mg/kg bw of DOX). As shown in Figure 5, blood DOX rapidly fell from 0.97 to 0.24 μg/mL within 10 hr of drug administration while blood ND-DOX slowly declined from 0.51 to 0.15 μg/mL over a course of 24 hr. Blood Plt@ND-DOX content gradually decreased from 1.67 to 1.12 μg/mL over a period of 16 hr but displayed a markedly higher value than the former two drugs at each and every sample point. Significantly slowed blood clearance is thus indicated of Plt@ND-DOX.

**Figure 6.**
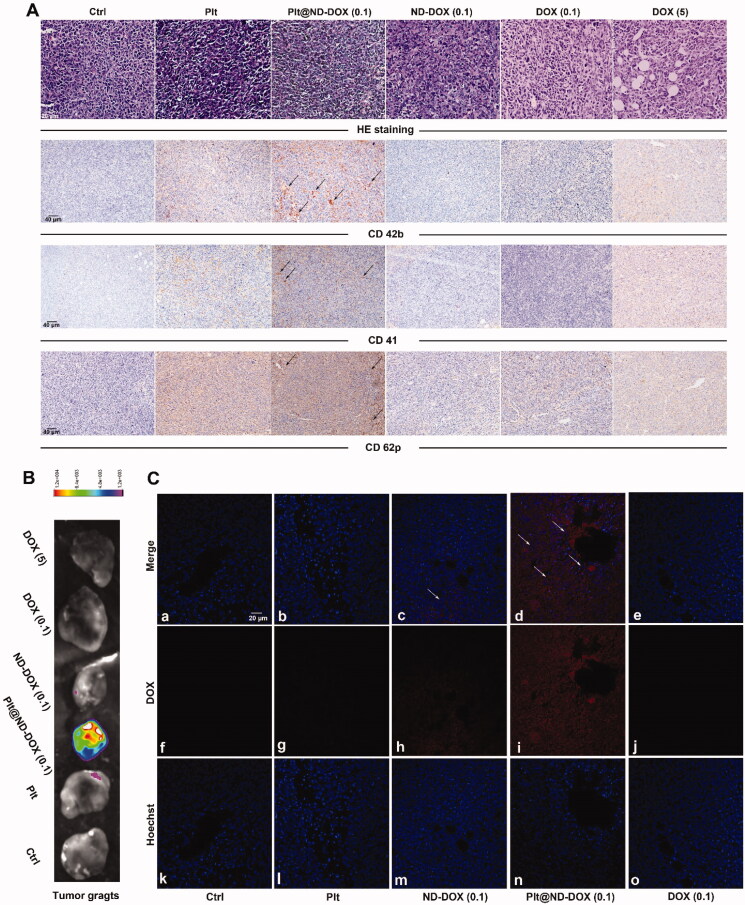
ND-DOX-loaded platelets infiltrated in tumor tissue and were activated to discharge Nano-DOX. (A) Graft tumors of Lewis lung cancer cells (LLC) that had received empty platelets or ND-DOX-loaded platelets displayed increased expression of markers of platelets (CD42b, CD41) and platelet activation (CD62p). (B) Ex vivo graft tumors that had received ND-DOX-loaded platelets exhibited pronounced DOX-derived fluorescence. (C) Fluorescent microscopy revealed extensive distribution of DOX-derived fluorescence in the tissue of graft tumors that had received ND-DOX-loaded platelets. Plt@ND-DOX refers to platelets loaded with ND-DOX; Plt@DOX refers to platelets loaded with DOX.

To corroborate the in vitro findings, mice bearing subcutaneous graft tumors of Lewis lung cancer cells were treated via intravenous injection with mouse platelets loaded with ND-DOX (Plt@ND-DOX) for 4 days quaque die. Loading condition was 300 × 10^6^ platelets incubated in 1 μg/mL of ND-DOX for 4 hr yielding a dose of 0.1 mg/kg bw in 200 μL of PBS. Injections of non-loaded platelets (Plt), ND-DOX (0.1 mg/kg bw) and DOX (0.1 and 5 mg/kg bw) were also given as controls. As shown in Figure 6(A), mice that received Plt or Plt@ND-DOX exhibited increased presence of platelets and platelet activation in the grafted tumors, as indicated by the elevated expression of CD 42 b, CD41 and CD62p. At the same time, only mice that received Plt@ND-DOX displayed pronounced doxorubicin-derived fluorescence in the graft tumors (Figure 6(B,C), Figure S3), indicating enhanced drug accumulation. Tumor drug accumulation was appreciable for ND-DOX but insignificant for DOX.

### 3.5. Nd-DOX-loaded platelets displayed pronounced anti-tumor potency with no apparent systemic and vital organ toxicity

**Figure 7.**
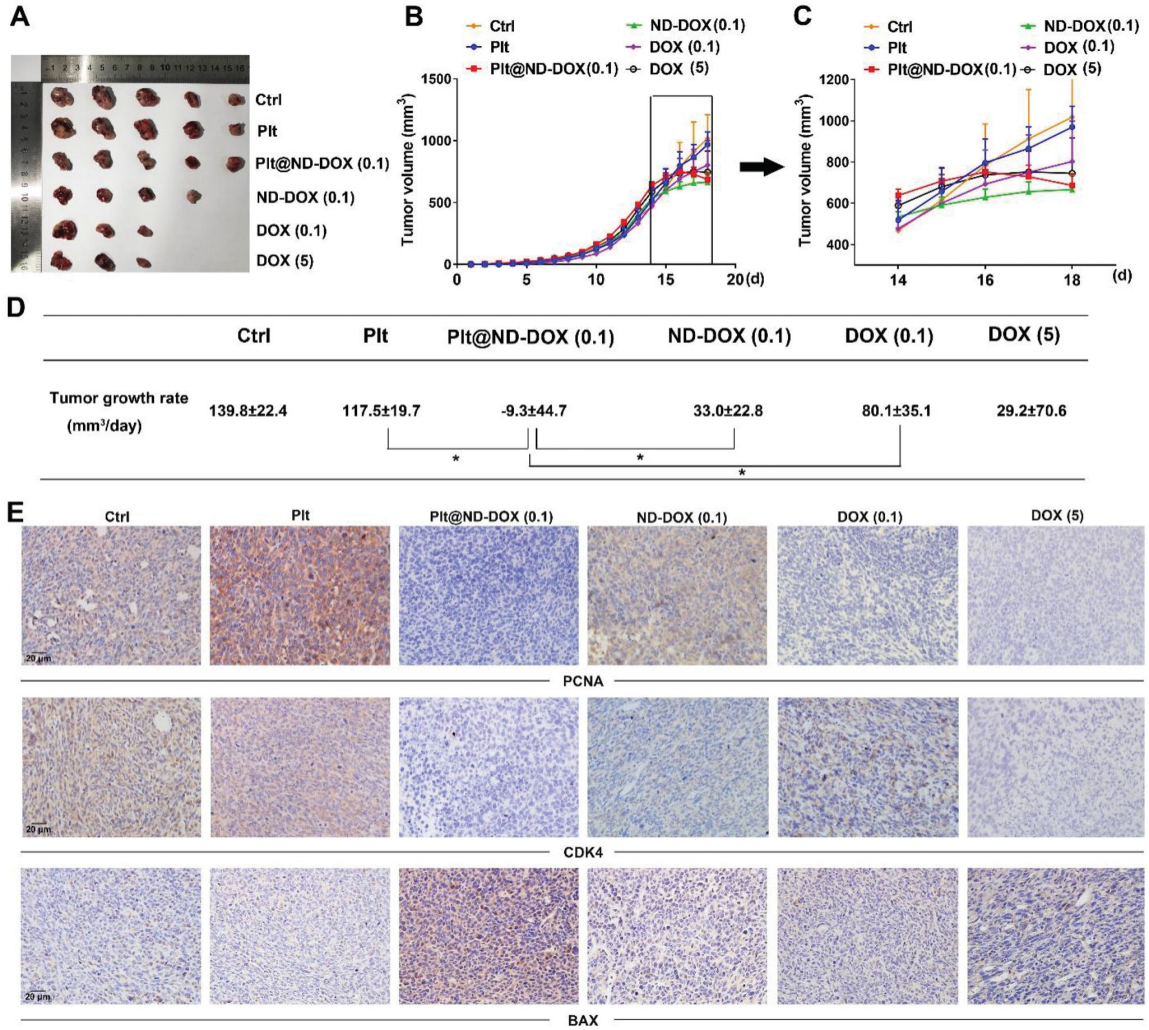
ND-DOX-loaded platelets exhibited high anti-tumor therapeutic potency. (A) Excised graft tumors of graft tumors of Lewis lung cancer cells (LLC). (B) Tumor growth from cancer cell inoculation until animal sacrifice. (C) Tumor growth over a 4-day treatment period from day 15 to day 18. The animals were sacrificed on day 18. (D) Tumor growth rates over the treatment duration (from day 15 to day 18) were calculated by regression analysis. (E) Expression of proliferation markers (PCNA and CDK4) and apoptosis marker BAX in the tumor tissues. Plt@ND-DOX refers to platelets loaded with ND-DOX; Plt@DOX refers to platelets loaded with DOX. Dose normalized to doxorubicin was 0.1 mg/kg bw for Plt@ND-DOX and ND-DOX. Doses for DOX were 0.1 and 5 mg/kg bw. Values were means ± SD (*n* ≥ 3, * *p* < .05).

**Figure 8.**
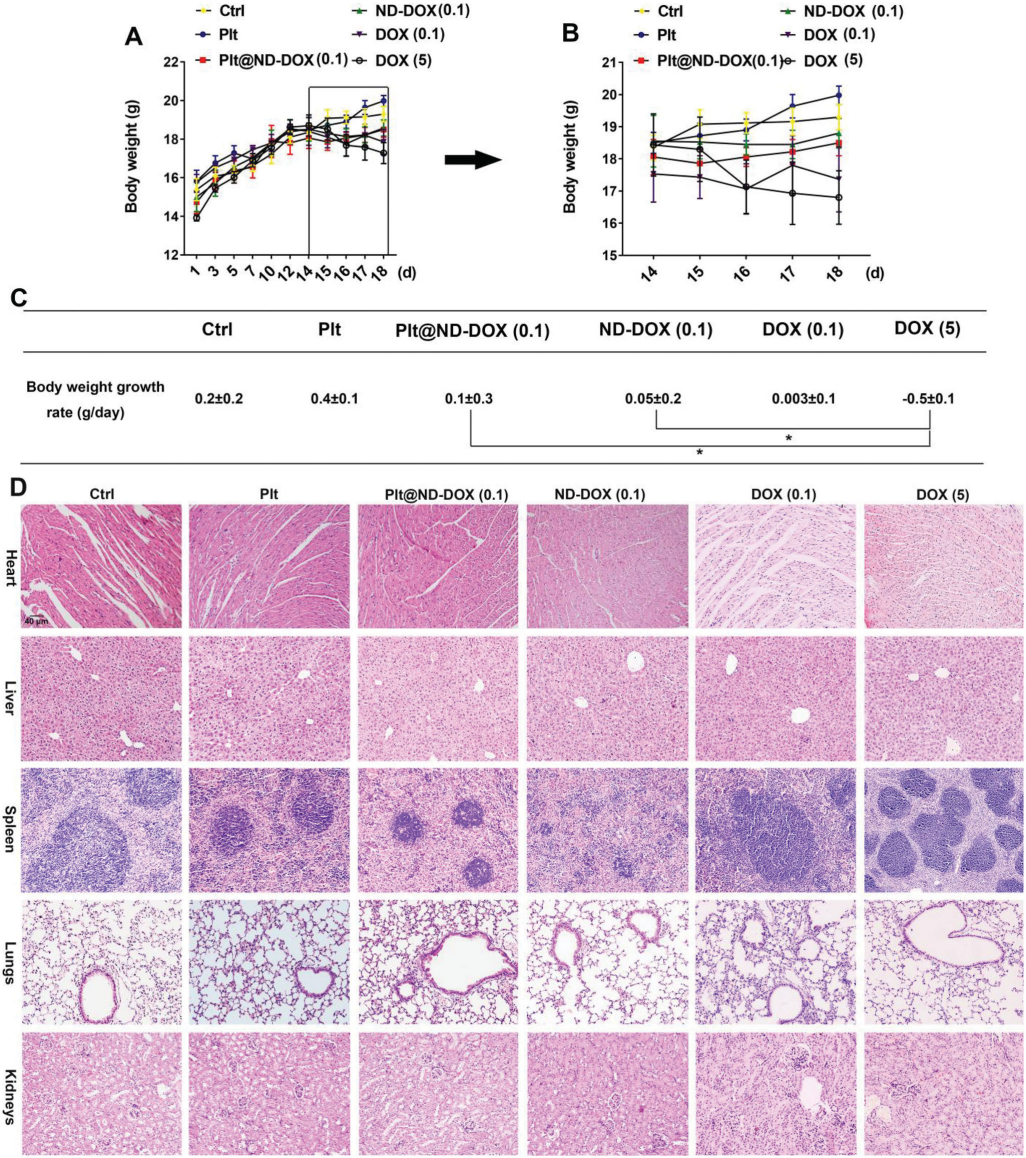
ND-DOX-loaded platelets exhibited no apparent systemic and vital organ toxicity. (A) Animal body weight growth from cancer cell inoculation until animal sacrifice. (B) Animal body weight growth over a 4-day treatment period from day 15 to day 18. The animals were sacrificed on day 18. (C) Body weight growth rates over the treatment duration (from day 15 to day 18) were calculated by regression analysis. (D) HE staining of vital organ tissues. Plt@ND-DOX refers to platelets loaded with ND-DOX; Plt@DOX refers to platelets loaded with DOX. Dose normalized to doxorubicin was 0.1 mg/kg bw for Plt@ND-DOX and ND-DOX. Doses for DOX were 0.1 and 5 mg/kg bw. Values were means ± SD (*n* ≥ 3, * *p* < .05).

In agreement with their drug delivery profile, Plt@ND-DOX (0.1 mg/kg bw) distinctly reversed the growth of Lewis graft tumors while the other treatments, i.e. ND-DOX at 0.1 mg/kg bw and DOX at 0.1 and 5 mg/kg bw, only slowed tumor growth to varied extents (Figure 7(A–C)). Consistently, Plt@ND-DOX markedly decreased the expression of proliferation markers PCNA and CDK4 and increased expression of the apoptosis marker BAX (Figure 7(D)) in the graft tumors. Importantly, while Plt@ND-DOX (0.1 mg/kg bw) exhibited a higher anti-tumor efficacy than 5 mg/kg bw of DOX, the severe systemic toxicity as indicated by a remarkable loss of body weight associated with DOX was not seen with Plt@ND-DOX. (Figure 8(A–C)). In consistence, mice that received Plt@ND-DOX (0.1 mg/kg bw) showed no apparent signs of vital organ damage (Figure 8(D)) and drug distribution (Figures S4 and S5). Note that mice treated with 5 mg/kg bw of DOX showed significant heart tissue inflammation (Figure 8(D)). It is worth noting that ND-DOX (0.1 mg/kg bw) also displayed remarkable therapeutic efficacy with a higher potency than DOX of the same dose but little sign of systemic toxicity (Figures 7(A–D) and 8(A–C)). Tumor weight and size also appeared to be the lowest in Plt@ND-DOX-treated mice at the experimental endpoint though the difference from other groups was not statistically significant likely due to small sample size (Supporting information Figures S6 and S7).

## 4. Discussion and conclusion

Targeted drug delivery is highly desirable for cancer therapy and platelets are gaining interest as a promising candidate for drug carrier serving this purpose. There are some apparent advantages to platelets as drug carriers. Platelets can naturally home to certain types of tissues, particularly cancer, and have a high loading capacity. From observations in our lab, platelet-mediated drug delivery minimizes the off-target action commonly seen with delivery devices based on nucleated cells e.g. monocytes, macrophages and neutrophils. Moreover, drugs delivered via platelets reduced the likelihood of being attacked by the immune system. Doxorubicin (DOX) is a first-line chemotherapeutic agent efficacious in the treatment of many malignant tumors. Collateral toxicity particularly to bone marrow and immune cells severely limit the clinical utility of DOX (Minderman et al., 1994; Fan et al., 2017). The present work explored the possibility and practicality of platelet-mediated tumor delivery of DOX in a nano-sized form i.e. ND-DOX. ND-DOX was DOX molecules attached to polyglycerol-coated nanodiamonds via the acid-cleavable hydrazone bond (Zhao et al., 2014). There have been reports of using platelets to deliver free DOX, but elucidation was lacking on the key issues of drug loading efficiency, capacity and particularly spontaneous drug release (Sarkar et al., 2013; Xu et al., 2017). In our work, we showed that ND-DOX could be loaded in mouse platelets in a dose-dependent manner with a markedly higher loading efficiency and capacity than free DOX. More importantly, loaded ND-DOX could be stably held in the platelets for at least 4 hr while loaded DOX displayed remarkable discharge within 4 hr of loading. This is also in contrast to nucleate cell-based carriers such as monocytes, macrophages and dendritic cells, which exhibited remarkable spontaneous unloading of ND-DOX (Li et al., 2017, 2018; Wang et al., 2018). Loading of ND-DOX appeared to have little impact on platelet viability; nor did the ND-DOX-loaded platelets (Plt@ND-DOX) show significant signs of activation. Induction of autophagy might have played a role in these observations as the internalized ND-DOX might be sequestered in the autophagosomes thus averting untoward happenings elsewhere. Induction of autophagy seems to be a universal cellular response to ND-DOX, which has also been observed in monocytes, macrophages, dendritic cells as well as tumor cells (Li et al., 2019). Interestingly, these immune cells are usually very sensitive to the toxicity of free DOX but platelets were found to tolerate DOX very well. The explanation for this discrepancy might be that DOX primarily acts to cause DNA damage in the nucleus, but platelets are anucleate thus lacking the target of DOX’s toxic action. The anucleate nature of platelets are particularly agreeable to the carriage of anti-tumor chemotherapeutic drugs, as the majority of them are cytotoxic agents having the nuclear DNA as the primary site of their actions. Cancer cells can activate platelets and activated platelets are known to release intracellular granules (Harrison & Cramer, 1993; Suzuki-Inoue, 2019; Zuo et al., 2019; Catani et al., 2020; Plantureux et al., 2020). It is on this ground that we had expected the mouse Lewis lung cancer cells would activate cargo unloading of Plt@ND-DOX, which was indeed confirmed in the co-culture experiments. Unsurprisingly, the unloaded ND-DOX was taken up by the co-cultured cancer cells which subsequently displayed viability loss, proliferation suppression and apoptosis. In previous studies, cancer cells were also observed to activate unloading of ND-DOX-loaded immune cells (Li et al., 2017, 2018; Wang et al., 2018).

The in vivo experiments corroborated the in vitro findings, confirming the infiltration, activation and cargo unloading of Plt@ND-DOX in the Lewis tumor grafts which displayed tumor regression, proliferation suppression and apoptosis. Due to low loading efficiency and spontaneous cargo unloading, DOX-loaded platelets were not evaluated in the in vivo study. Instead, Plt@ND-DOX was evaluated against ND-DOX and free DOX for comparison. The therapeutic efficacy of Plt@ND-DOX did not appear a surprise to us. What surprised us is the remarkable potency of Plt@ND-DOX (0.1 mg/kg bw) which effectively reversed the tumor growth while ND-DOX (0.1 mg/kg bw) and DOX even at a dosage as high as 5 mg/kg bw only slowed tumor growth. It must be noted that ND-DOX largely lacks the tumoricidal activity of free DOX (Gresele et al., 2017; Li et al., 2018), which renders the high anti-tumor potency of Plt@ND-DOX even more impressive and intriguing. Moreover, while Plt@ND-DOX (0.1 mg/kg bw) exhibited a higher therapeutic efficacy than 5 mg/kg bw of DOX, the severe systemic toxicity associated with DOX was strikingly absent with Plt@ND-DOX. The remarkably high therapeutic potency and absence of collateral normal tissue damage of Plt@ND-DOX, we posit, may be largely attributed to the high tumor targeting property and the long blood circulation time (indicated by slow clearance) of the platelet carriers, giving rise to greatly enhanced drug accumulation and retention in the tumors. Concerning the tumor-targeting property of Plt@ND-DOX, there might be two possible mechanisms. First, spontaneous intratumor hemorrhage, which are common in solid tumors, may lead to leakage of Plt@ND-DOX in the tunro tissue. Second, platelets are known to release their granular components in the tumor vasculature to stabilize micro vessels (Ho-Tin-Noé et al., 2009). Plt@ND-DOX may unload drug cargo via the same process in the tumor vasculature. Third, platelets are capable of migration and extravasation and they can be activated by the cancer cells to degranulate, which can also happen to Plt@ND-DOX. Furthermore, ND-DOX is able to stimulate the tumor cells to emit DAMPs which are potently inflammatory (Li et al., 2017, 2018) and may enhance the aforementioned behavior of platelets and Plt@ND-DOX thus potentiated tumor targeting. It is also worth mentioning that platelets have been reported to exert cytotoxic action against cancer cells and to promote anti-cancer immunity (Gresele et al., 2017; Miao et al., 2020). Whether these elements are involved in the high therapeutic potency of Plt@ND-DOX is an interesting question worthy of further investigation.

One last point for discussion is that we plotted the tumor growth curves, from which the tumor growth rates were calculated and compared among different treatment groups to show therapeutic potency. This approach is more rigorous than merely comparing the tumor weight and size at the experimental endpoint. The latter approach (comparing the tumor weight and size at the experimental endpoint) might be misleading as all tumors were not at the same weight or size when the treatment was started. It may well be possible that tumors subjected to a treatment both start and end with a bigger size/weight than control tumors, but still display a slower growth rate. For reference, data of tumor weight and size at the experimental endpoint are provided as supporting information (Figures S6 and S7).

**Figure 9.**
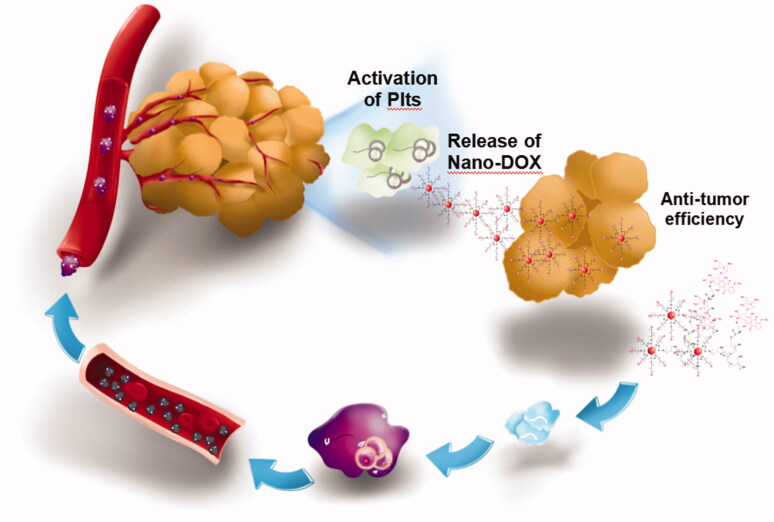
The overview of the present study

To summarize, our findings serve as concrete proof for the concept of platelet-mediated nano-drug delivery which has the following features: 1) easy preparation of platelets, 2) large loading capacity and high loading efficiency, 3) good tolerance of cargo drugs, 4) stable cargo retention and no cargo unloading in the absence of stimulation, 5) prolonged blood circulation time, and 6) excellent tumor distribution and tumor-activated drug unloading leading to high therapeutic potency and few adverse effects (Figure 9).

## Supplementary Material

Supplemental Material
