# Supplementary material for: Platelets are highly efficient and efficacious carriers for tumor-targeted nano-drug delivery
Source: Drug Deliv. 2022 Mar 23;29(1):937–49. doi: 10.1080/10717544.2022.2053762 (PMC8956315; doi:10.1080/10717544.2022.2053762)
Supplement: Supplemental Material [file IDRD_A_2053762_SM8470.docx]

Platelets are highly efficient and efficacious carriers for tumor-targeted

nano-drug delivery

Qi-Rui Li ^a,b,#^, Hua-Zhen Xu ^c,#^, Rong-Cheng Xiao ^a,#^, Yan Liu ^c^, Jun-Ming Tang ^a^, Jian Li ^a^, Ting-Ting Yu ^a,b^, Bin Liu ^a,b^, Liu-Gen Li ^a,b^, Mei-Fang Wang ^b^, Ning Han ^a,b^, Yong-Hong Xu ^d^, Chao Wang ^c^, Naoki Komatsu ^f^, Li Zhao ^e^, Tong-Fei Li ^a,b^, Xing-Chun Peng ^a,b,*^, Xiao Chen ^c,*^

^a^ School of Basic Medical Sciences, Hubei University of Medicine, Renmin road No. 30, Shiyan, Hubei, 442000, China.

^b^  Hubei Key Laboratory of Embryonic Stem Cell Research, Taihe Hospital of Shiyan, Hubei University of Medicine, Renmin road No. 30, Shiyan, Hubei, 442000, China.

^c^ Department of Pharmacology, School of Basic Medical Sciences, Wuhan University, Donghu Avenue No.185, Wuhan 430072, China; Hubei Provincial Key Laboratory of Developmentally Originated Disease, Wuhan 430071, China.

^d^ Institute of Ophthalmological Research, Department of Ophthalmology, Renmin Hospital of Wuhan University, Wuhan 430060, China.

^e^ State Key Laboratory of Radiation Medicine and Protection, School of Radiation Medicine and Protection & School for Radiological and Interdisciplinary Sciences (RAD-X), Collaborative Innovation Center of Radiation Medicine of Jiangsu Higher Education Institutions, Soochow University, Suzhou, Jiangsu 215123, China.

^f^ Graduate School of Human and Environmental Studies, Kyoto University, Sakyo-ku, Kyoto 606-8501, Japan

* E-mail address: [Litongfeihappy@163.com](mailto:Litongfeihappy@163.com) (Tong-Fei Li), chen-xiao-1976@hotmail.com (Xiao Chen);

^#^These authors contribute equally to this article

**Supporting information:**


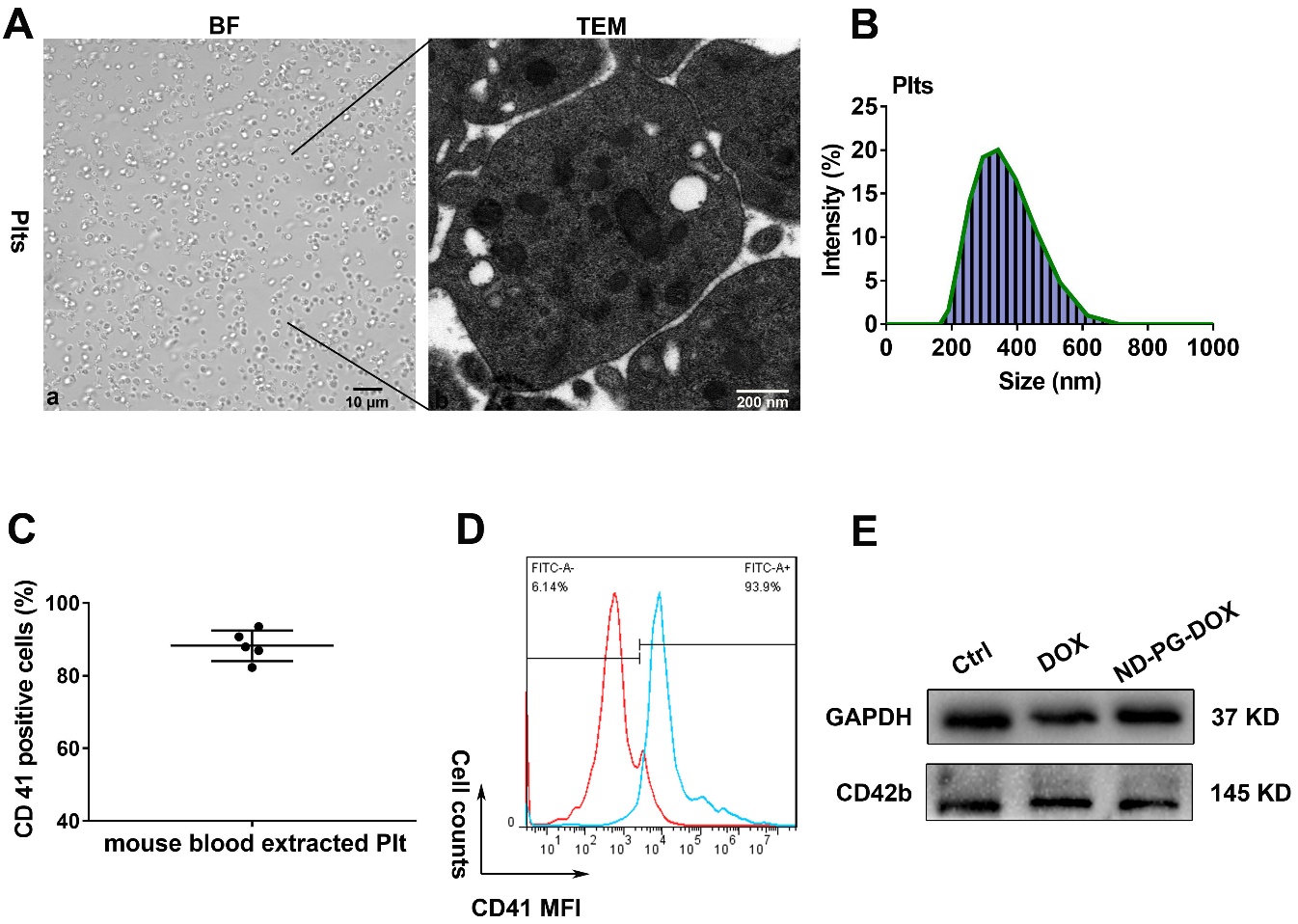


**Fig. S1.** **Characterization of mouse platelets (Plt).** **A:** Platelets were observed using confocal microscopy and TEM. **B:** The size of platelets was detected by DLS. **C-D:** Surface expression of CD41 in platelets was assayed using immunofluorescent staining and flow cytometry. The CD41 positive platelets were computed. **E:** Expression of CD42b which is a biomarker of platelets was assayed using western blotting.

**
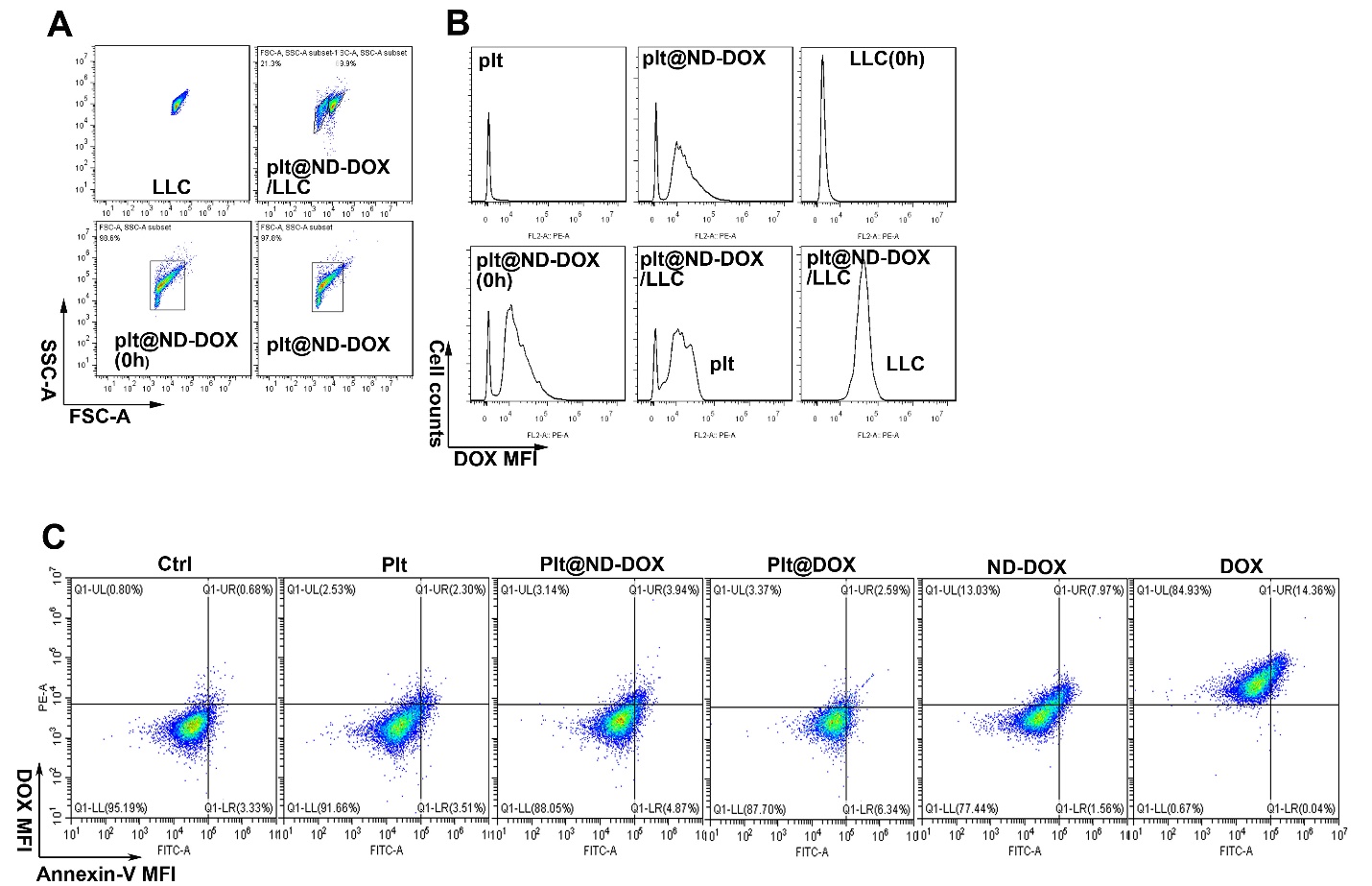
**

**Fig. S2. Representative flow cytometry raw data for Fig. 4 F, G, & K.**


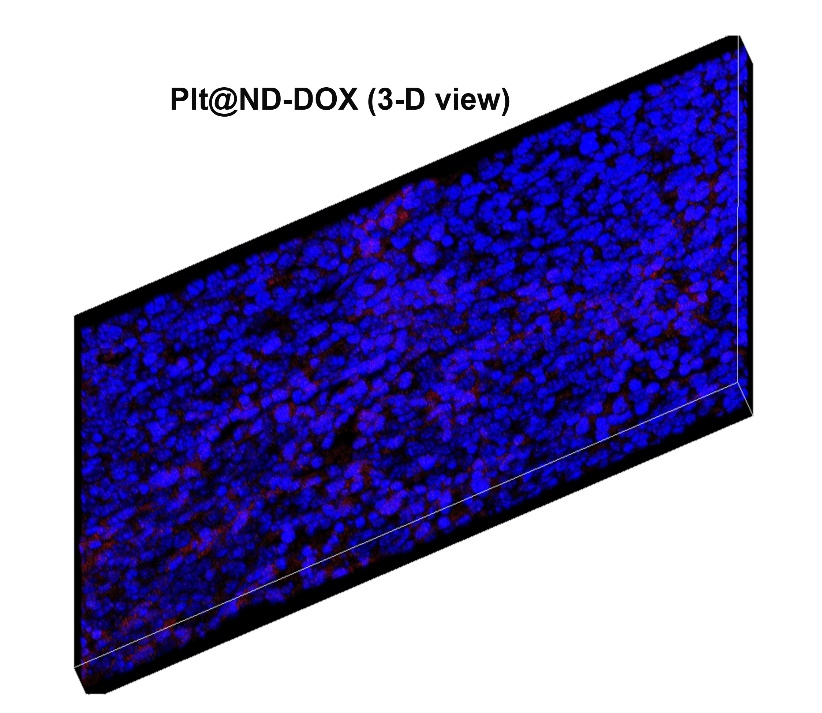


**Fig. S3. 3-D view of tumor graft tissue that was treated with ND-DOX-loaded platelets.**

**
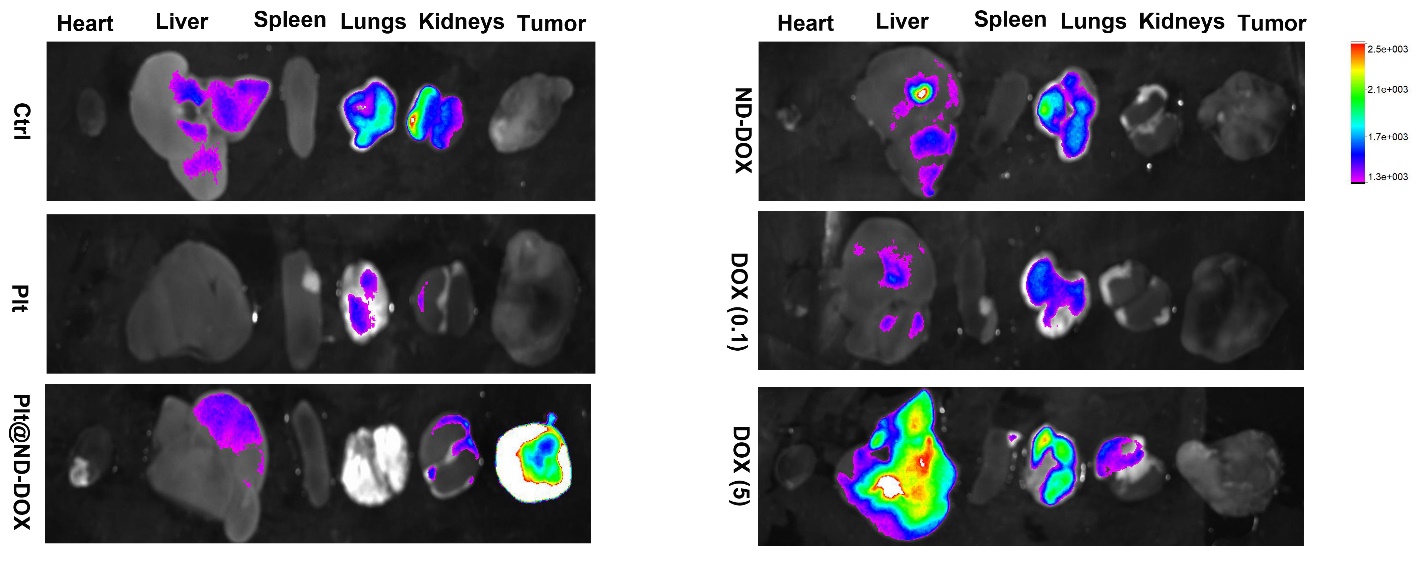
**

**Fig. S4. Fluorescent imaging of ex vivo vital organs from mice that received different treatments.**

**
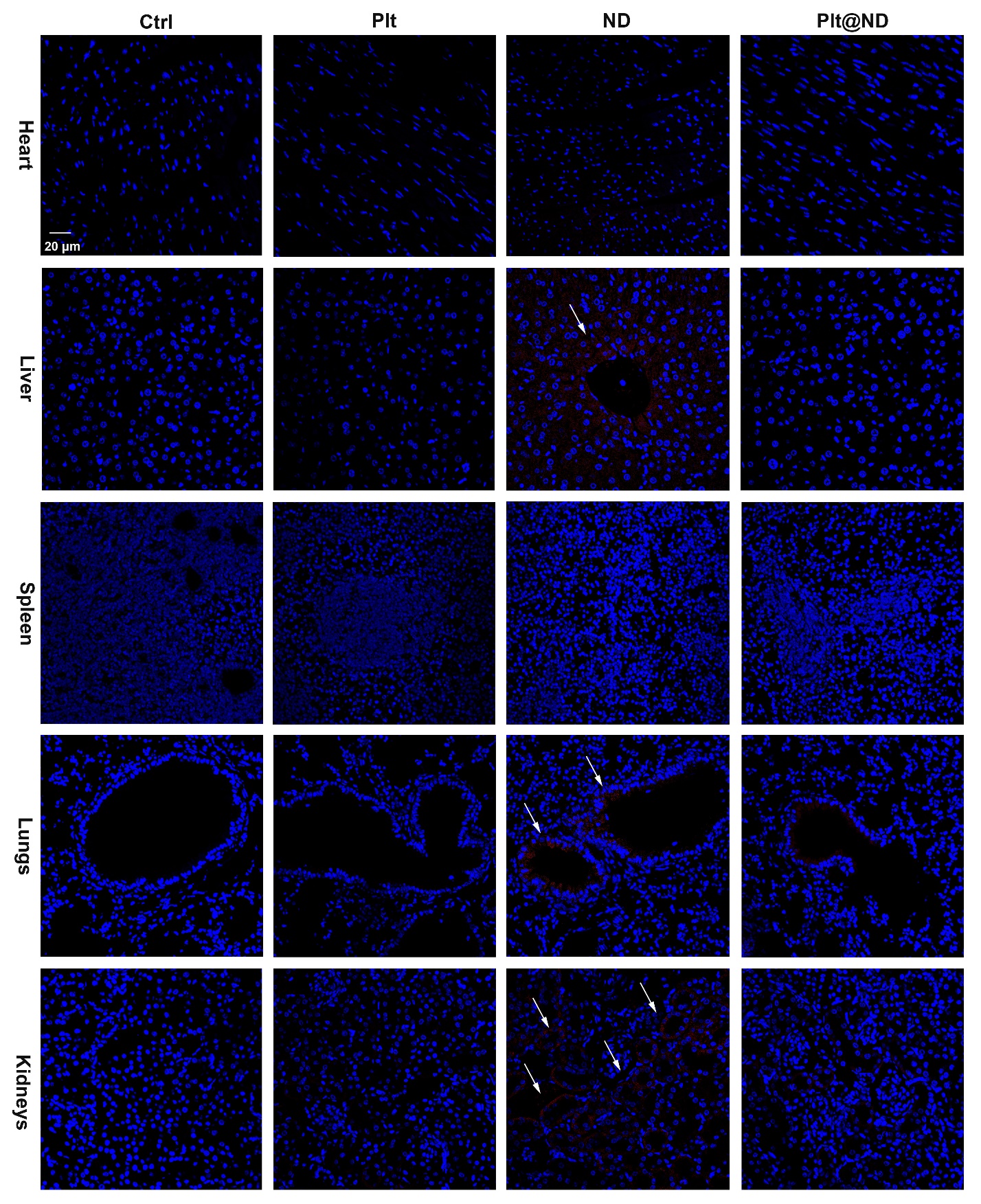
**

**Fig. S5.** **Distribution of ND-DOX in vital organs of mice that were treated with ND-DOX-loaded platelets.** Organ tissue slides were subjected to immunofluorescent staining and observed using confocal microscopy. Blue fluorescence came from nucleus staining. Red fluorescence came from ND-DOX. Arrow marked were sites of ND-DOX.


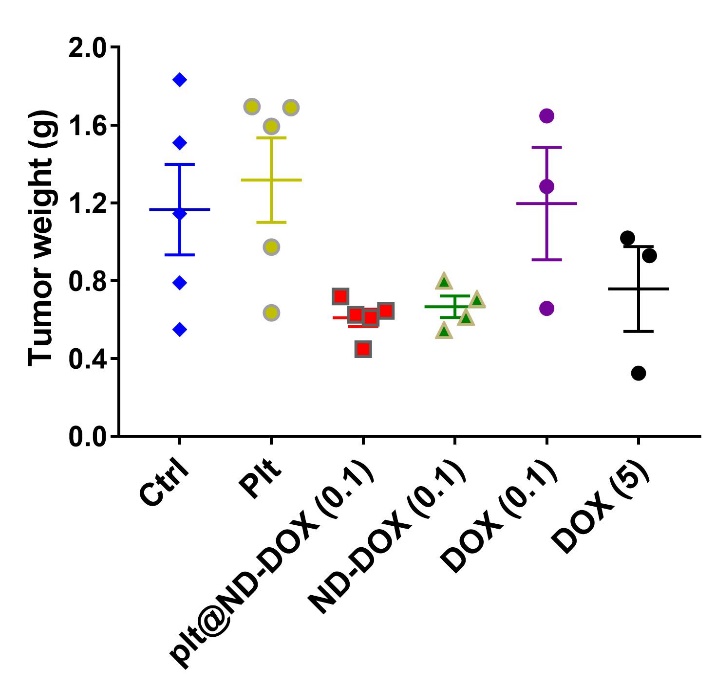


**Fig. S6. Tumor weight at the experimental endpoint (day 18). Values were means ± SD (n ≥ 3).**

**
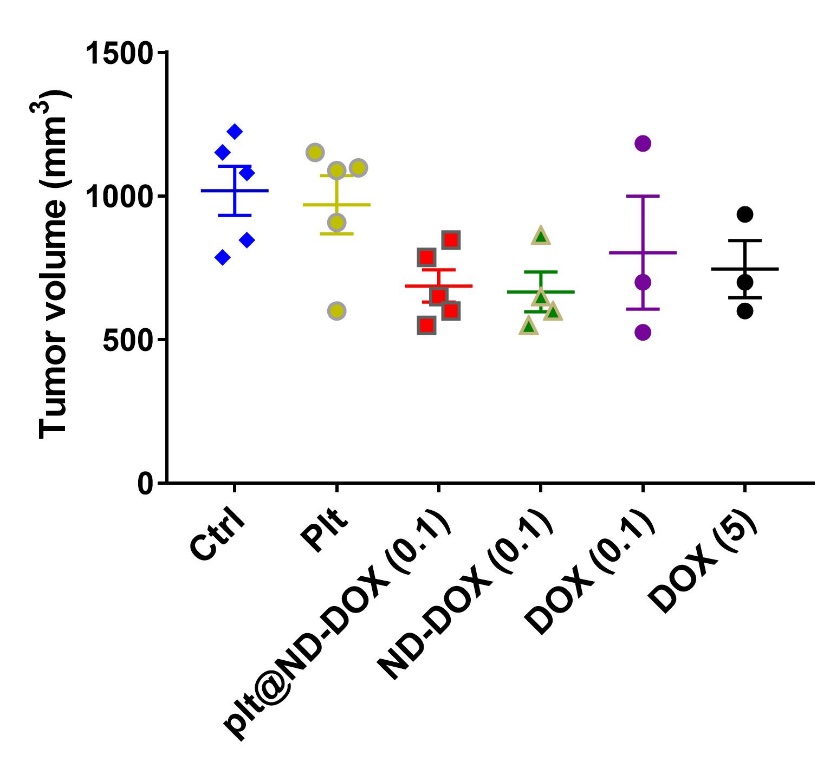
**

**Fig. S7. Tumor size at the experimental endpoint (day 18). Values were means ± SD (n ≥ 3).**
